# Radiographic Analysis in Extra-Articular and Intra-Articular Distal Radius Fractures Treated with Variable-Angle Volar Locking Plate Fixation

**DOI:** 10.3390/jcm12103494

**Published:** 2023-05-16

**Authors:** Pin-Chieh Fang, Tak-Yu-Yubie Lo, Chun-Ying Cheng, Chun-Te Wu, Alvin Chao-Yu Chen

**Affiliations:** 1 Chang Gung Memorial Hospital-Linkou, Chang Gung University College of Medicine, Taoyuan 33305, Taiwan; andy40640@gmail.com (P.-C.F.); yubie.lty@gmail.com (T.-Y.-Y.L.); 2 Bone and Joint Research Center, Department of Orthopaedic Surgery, Chang Gung Memorial Hospital-Linkou, Chang Gung University College of Medicine, Taoyuan 33305, Taiwan; orthhand@cgmh.org.tw; 3 Comprehensive Sports Medicine Center, Chang Gung Memorial Hospital-Taoyuan, Chang Gung University College of Medicine, Taoyuan 33305, Taiwan; 4 Department of Medical Imaging and Intervention, Chang Gung Memorial Hospital-Linkou, Chang Gung University College of Medicine, Taoyuan 33305, Taiwan; melik@cgmh.org.tw

**Keywords:** distal radius fracture, variable-angle volar locking plate, radiographic parameters

## Abstract

(1) Background: Different distal radial fracture types have different prognosis after fixation. Our study aim is to evaluate the differences in radiographic parameters by using variable-angle volar locking plate (VAVLP) fixation according to extra-articular and intra-articular distal radial fracture. (2) Methods: There are two groups: extra-articular group (21) and intra-articular group (25). Forearm radiographs immediately after surgery and at 3 months after operation were reviewed for analyzing radial height (RH), ulnar variance (UV), radial inclination (RI), volar tilt (VT), tear drop angle (TDA), distal dorsal cortical distance (DDD), and Soong classification (SC). (3) Results: There were no significant differences in the above parameters between two groups at either immediately post-operative or at 3-month follow-up, except for TDA (*p* = 0.048). Most patients in both groups were at low risk of flexor tendon rupture, except for two cases. We observed a positive correlation between post-operative DDD and 3-month change in the intra-articular group, but not the extra-articular group. (4) Conclusions: Our study demonstrates that VAVLP fixation is effective in maintaining the stability of most radiographic parameters and reduces the risk of tendon rupture in both extra-articular and intra-articular distal radial fractures. Post-operative DDD can be used to predict the degree of subsequent displacement in patients with intra-articular fractures fixed with VAVLP.

## 1. Introduction

Distal radial fracture is a prevalent injury in our field, and its incidence has been on the rise [1,2]. Despite the ongoing advancements in medical technology, there is no one-size-fits-all treatment modality for displaced distal radius fractures. In light of recent trends, surgery with internal fixation using a volar locking plate (VLP) has become the preferred option over percutaneous fixation for distal radial fractures with displacement [3,4].

Within the category of volar locking plates (VLP), there are two types distinguished by their locking mechanisms: fixed-angle volar locking plates (FAVLP) and variable-angle volar locking plates (VAVLP). A traditional FAVLP fixation can enhance stability by creating a rigid construct with screws inserted and locked in a predetermined direction without taking into account the fracture’s personality or any variability in plate positioning. For some comminuted fractures, the use of additional fixation methods such as Kirschner wire was required [5,6]. However, VAVLP has been shown to be more effective in treating complex intra- and extra-articular fractures that involve the watershed line and volar rim due to its greater screw angle insertion adaptability. Additionally, when compared to the traditional FAVLP, VAVLP has been associated with fewer reported complications such as symptomatic hardware, loose hardware, malunion and tendon rupture [6,7]. 

In the field of biomechanics, there have been numerous studies on the stability of VAVLP, as well as studies investigating the use of VAVLP in treating Colle’s fracture and Smith’s fracture in adult patients [8,9,10]. However, when it comes to classifying fractures, injury patterns that involve the articular surface may provide a more direct reflection of radiographic parameters and prognosis. Despite this, there is limited clinical research on the statistical analysis of radiographic parameters following the use of VAVLP for different type of distal radial fractures. Most of the studies in the past decade have focused on traditional radiographic parameters such as radial height, ulnar variance to evaluate the stability after volar locking plate fixation [4,6,7,10]. Nevertheless, in recent years, new radiographic parameters such as Soong classification and distal dorsal cortical distance have been proposed. 

The objective of this study is to compare traditional and modern radiographic parameters following the use of variable-angle volar locking plates (VAVLP) for both extra-articular and intra-articular distal radial fractures. Our evaluation will include measurements taken immediately after the operation as well as at the 3-month postoperative follow-up.

## 2. Materials and Methods

### 2.1. Research Design and Planning

This retrospective research involved patients with distal radius fracture who underwent open reduction and internal fixation (ORIF) with VAVLP between October 2011 and October 2022 at a tertiary trauma center. The Institutional review board approved this study (IRB 202000939B0). 

The inclusion criteria comprised all types of distal radial fractures diagnosed by forearm X-ray using Arbeitsgemeinschaft für Osteosynthesefragen/Orthopaedic Trauma Association (AO/OTA), and patients who underwent VAVLP fixation (DePuy Synthes, 2.4 mm Variable Angle LCP) [11]. All patients were adults, with the oldest being 86 years of age. Patients who left follow-up during the evaluation stage and radiographs with poor quality were excluded from the study.

An experienced hand surgeon (Chen, ACY) performed all surgical procedures. All patients with unilateral distal radial fracture underwent VAVLP fixation and were fitted with a short-arm splint for postoperative protection for six weeks. To evaluate the stability of VAVLP in radial fracture fixation, posteroanterior (PA) and lateral views of forearm X-ray were measured immediately after the operation and at the 3-month follow-up [11,12]. We confirmed that all fractures had healed after three months, and there were no instance of infection or revision surgery. Previous research indicated that three months was sufficient for radiographic healing of distal radial fractures, which was evidenced by bridging callus or osseous bone [13]. All electronic medical records and diagnostic imaging studies were assessed by two of co-authors (Fang, PC and Chen, ACY) with a consensus regarding the assessment.

### 2.2. Analysis of Forearm X-ray Images

**Figure 1:**
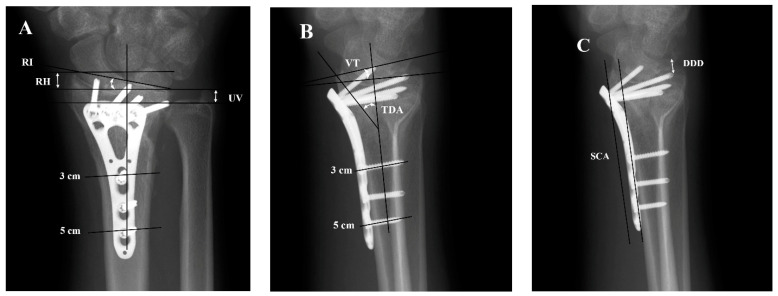
Radiographs of posteroanterior (**A**) and lateral view (**B**,**C**) of distal radius fracture with variable-angle volar locking plate. RH = Radial height, UV = Ulnar variance, RI = Radial inclination, VT = Volar tilt, TDA = Tear drop angle, DDD = Distal dorsal cortical distance, SCA = Soong classification auxiliary line.

The X-ray images of distal radial fracture patients were divided into two groups, extra-articular and intra-articular, and radiographic parameters were measured [9]. To ensure standardization, radiographic parameters were measured on standard wrist X-rays using the methods introduced by Kreder et al. [14]. On both lateral and PA views, the center of the radius was marked at 3 and 5 cm below the midline of the proximal articular surface of the lunar bone to form the radial central axis [14]. Radial height, ulnar variance, radial inclination were measured on the PA view [15], while volar tilt, teardrop angle (TDA), distal dorsal cortical distance (DDD), and Soong classification (SC) were measured on the lateral view. TDA is the angle between a line perpendicular to the central axis and a line that passes through the central axis of the teardrop [16,17]. SC is defined based on a tangential line drawn to the volar rim, parallel to the diaphyseal bone of the radial shaft, to determine the plate prominence. Plates that do not exceed the line were documented as Grade 0; plates that are palmar to the line but remain proximal to the rim were documented as Grade 1; plates that were directly on or distal to the volar rim were documented as Grade 2 [18]. DDD refers to the distance between the dorsal rim of the distal end of the radius and the tip of the most distal screw [19] (Figure 1). A radiologist (W, C-T) who was blinded to surgery confirmed all radiographic analyses.

The radiographic parameters mentioned above were measured using the PAC computer program, PACS with a high degree of accuracy (0.01 mm and 0.1). 

### 2.3. Statistical Analysis

Mann–Whitney U test and Fisher’s exact test were applied to compare the characteristics of patients in extra-articular and intra-articular groups. 

In order to analyze the data collected, the following statistical tests were used by the orthopedic specialists:

- Paired *t*-tests and Wilcoxon signed-rank tests were used to compare radial height (RH), ulnar variance (UV), radial inclination (RI), volar tilt (VT), teardrop angle (TDA), and distal dorsal cortical distance (DDD) immediately after the operation and 3 months following the operation within each of the two groups.
- Student’s *t*-tests were used to compare RH, UV, RI, VT, TDA, and DDD immediately after the operation with 3-months follow-up in the extra-articular and intra-articular groups, as well as to compare the 3-month change in RH, UV, RI, VT, TDA, and DDD between these two groups.
- The Soong classification gradings were divided into low-risk (Grade 0) and high-risk (Grade 1 and Grade 2) groups to analyze the relationship between the risk of flexion tendon rupture and types of fracture using the likelihood Chi-square test and Fisher’s exact test.
- Linear regression was used to analyze the DDD estimated immediately after the operation and the change at 3-months follow-up in both the extra-articular and intra-articular groups, as well as in all patients in both groups.

All statistical analyses were conducted with SPSS v21 (IBM Corporation, Armonk, NY, USA).

## 3. Results

### 3.1. Characteristics in Study Subject

We enrolled 46 patients who received VAVLP fixation for distal radial fracture and classified them into two groups: extra-articular group (21 patients) and intra-articular group (25 patients). There is no significant difference in age, timing of imaging immediately after operation, timing of imaging at 3-month follow-up, gender, and dominant side distribution between the two groups (Table 1).

### 3.2. Radiographic Parameters of Immediately Post-Operation and 3 Months following Operation in Extra-Articular Group and Intra-Articular Group

There is no statistically significant difference in parameters between the extra-articular and intra-articular groups in terms of most radiographic parameters immediately after the operation and at 3-month follow-up. However, TDA showed a significant difference between the two groups immediately after operation, with a *p*-value of 0.048. The parameters of RH, UV, RI, VT, and TDA of both groups immediately after the operation and at 3-month follow-up did not show any significant difference, with *p*-values above 0.05. On the other hand, the DDD value of intra-articular group immediately after the operation and at 3-month follow-up showed a significant difference with a *p*-value of 0.008 (Table 2).

### 3.3. Comparison of Risk of Tendon Rupture by Soong Classification at Two Timings and Change of Tendon Rupture Risk in 3 Months between Extra-Articular Group and Intra-Articular Group

No significant difference was found between the extra-articular and intra-articular groups in terms of flexor tendon rupture risk at both post-operation and 3 months following the operation, with *p* = 0.585 and *p* = 0.443, respectively. The majority of patients in both groups belonged to the low-risk group for flexor tendon rupture, regardless of post-operation or three months after operation. However, two cases exhibited upgrades from the low-risk group to the high-risk group at 3 months, although there was no significant difference with a *p*-value of 0.225 (Table 3).

### 3.4. Linear Regression of DDD of Immediately Post-Operation and Changes in 3 Months following the Operation in the Extra-Articular Group, Intra-Articular Group and All- Patients

Linear regression and curve estimation were used to compare DDD right after the operation and the change in DDD after 3 months following the operation in the extra-articular group, intra-articular group, and the combined group of both. The results showed that there was a significant difference between the intra-articular group and the combined group, as well as the extra-articular group having no significant dependence (Table 4).

**Figure 2:**
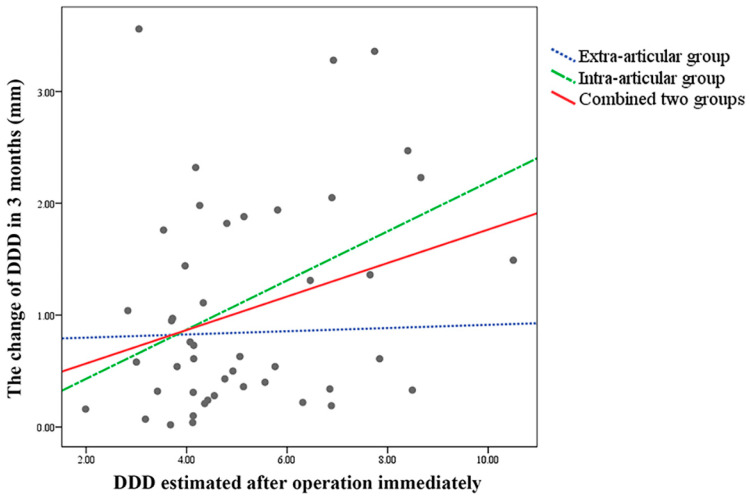
Linear regression of change in DDD of the extra-articular group, intra-articular and group combining the above two groups immediately after the operation and 3 months following the operation. DDD = distal dorsal cortical distance.

The *p*-values for the intra-articular group and the combined group were 0.031 and 0.048, respectively. The scatter plot and B value of linear regression revealed that the DDD immediately post-operation had a greater impact on the change in DDD after 3 months following the operation in the intra-articular group compared to the extra-articular group and the combined group (Figure 2).

## 4. Discussion

VAVLP has gained popularity as a first-line treatment for distal radial fractures in ORIF. Several studies have suggested that VAVLP can effectively maintain radiographic and postoperative outcomes. However, most studies have not analyzed cases based on fracture severity, such as fracture types. The fluctuating proportion of extra-articular fractures may lead to overestimation of the efficiency of VAVLP. In a study by Sherif Elerian et al., it was demonstrated that VAVLP showed good reduction on radiographs and only mildly restricted mean ranges of movement. The study included 61 patients with distal radius fractures consisting of 19 AO type A, 9 AO type B, 33 AO type C fractures [20]. 

Our study compared radiographic parameters immediately after operation and at 3-month follow-up as well as at different checkpoints, between two groups. In addition to commonly used parameters such as RH, UV, RI, and VT, we also included the concept of TDA [17], which is particularly useful in evaluating displaced intra-articular fracture and incongruity of the volar lunate facet. This allowed a more comprehensive reference for radiographic parameters. Our results suggested that VAVLP can effectively maintain radiographic parameters, both extra-articular and intra-articular. While there were notable variations in TDA values following the surgery between the two groups, we postulated that the principal contributor to this discrepancy is likely the presence of observation errors within the statistical distribution of the data. 

One previous study had found that VAVLP could maintain radiographic parameters more effectively than FAVLP in treating intra-articular and extra-articular distal radial fractures [21]. Otherwise, another study by Erhart et al. in 2018 reported decreased radial inclination and volar tilt in Colle’s type distal radial fractures fixed with FAVLP but no significant change in radiographic parameters for Smith type distal radial fractures fixed with FAVLP [10]. However, they did not specify a clear reason for this difference. Another retrospective study involving 7 type A3, 8 type C2, and 18 type C3 distal radius fractures fixed with FAVLP found no significant difference in radiographic parameters between post-operation and 1-year follow-up [22]. However, the evaluation for the ability of FAVLP fixation may have been overrated and blurred by the inclusion of AO classification type A3. We presume that the type of distal radial fracture could affect the effectiveness of fixation with FAVLP when evaluated using traditional parameters.

In a prior investigation, 200 images of normal wrists and 95 images of wrists with distal radial fractures were collected and evaluated for TDA value. The findings indicated that the average TDA value for normal wrists was 68 degrees, while injured wrists after reduction had an average TDA value of 58 degrees, respectively [23]. In our own research, by comparing the two groups, we compared the TDA values of extra-articular and intra-articular groups and found that the former is much closer to the mean TDA value of injured wrists after reduction. Additionally, no discernible difference was observed in TDA value between the immediate post-operation stage and 3-month follow-up in either group. Another previous study corroborated our result demonstrating no significant changes in TDA value during 3 months under fixation with VAVLP [21]. Unfortunately, there has been no research utilizing TDA to evaluate the long-term stability of the lunate surface in FAVLP.

Soong M et al. reported a stronger association between flexor tendon rupture and grade 1 and grade 2 of the Soong classification [19,24]. Moreover, the likelihood of removing the VLP due to post-operative complications of grade 1 and grade 2 was 1.5 and 6 times higher, respectively, than grade 0. In a 2020 investigation by Yekta Gören, flexor tendon rupture was observed exclusively in patients classified as grade 1 and grade 2, and it was recommended that these patients consider VLP removal after the recovery period [25]. Based on these factors, we have categorized grade 0 as low-risk group and grades 1 and 2 as high-risk groups for this study. 

The findings of our study revealed that a majority of cases in both extra-articular and intra-articular groups were classified as low-risk of flexor tendon rupture at the immediate post-operation stage. This outcome could be attributed to the design of VAVLP with an anatomic shape that fits close to the volar ridge, and rounded edges that minimize soft tissue irritation. Caroline A Selles et al. conducted a study in 2018 to analyze the distribution of Soong classification gradings in patients who underwent FAVLP fixation. The study included 323 cases, with 28%, 58%, and 14% as grades 0, grade 1, grade 2, respectively [26]. In our study, the percentage of patients classified as grade 2 immediately after the operation was 10%, which decreased to 7% at the 3-month follow-up. The percentages at both checkpoints were lower than those reported in the after mentioned study. Additionally, a study by Yamak K et al. in 2018 that employed VAVLP fixation demonstrated that the percentage of patients classified as grade 0, grade 1, and grade 2 were 72.4%, 21.1%, and 6.5%, respectively [27]. These results are similar to those of our study and indicate that VAVLP fixation poses a lower risk of flexor tendon rupture compared to traditional FAVLP fixation. 

Our analysis did not reveal any significant difference in the Soong classification immediately after the operation and at the 3-month follow up in both the extra-articular and intra-articular groups. However, two cases in the intra-articular group had a shift from low-risk to high-risk. Upon further review, we found that both cases belonged to AO classification 2R3C1. In this fracture type, the distal articular surface is broken by the transverse fracture line, and the distal dorsal fragment splits into half in sagittal plane. Under such situation with severe intra-articular comminution, VAVLP may not entirely eliminate the risk of tendon rupture. Nevertheless, VAVLP has a good ability to reduce the Soong gradings for most fracture types. 

The concept of using the measurement of DDD to optimize screw and plate fixation was first introduced in 2015, and it was recommended that plate fixation should not exceed 6 mm in maximum during surgery is recommended to prevent subsequent displacement [18]. When a positive correlation exists between the DDD immediately after the operation and the change in DDD at the 3-months follow-up, it may serve as a predictor for the degree of subsequent displacement. 

Our study found that only the intra-articular group and combined group revealed a positive correlation between the change of DDD value, making them the only groups where DDD can be used as a predictor of subsequent displacement. Previous research by Sang KL and colleagues showed that if the distance between the tip of the most distal screw and the dorsal rim of the distal radius is too short, the predictability of post-operative DDD decreases [28]. In our study, the average of post-operation estimated DDD values were 4.69 and 5.55 in the extra-articular and intra-articular groups, respectively. The minimum of post-operation estimated DDD value were 1.99 and 3.42 in the extra-articular and intra-articular groups, respectively. However, the variation in DDD values over 3 months was only 0.84 and 1.21 in the extra-articular and intra-articular groups, respectively. We assumed that while DDD was not an effective predictor in the extra-articular group, the subtle 3-month variation in DDD values did not affect the fixation stability. On the other hand, using the post-operation estimated DDD value to predict the degree of subsequent displacement was feasible. 

There are several limitations to our study. Firstly, the radiographic analysis is based on a retrospective case review without a control group, although there is no significant difference in patient characteristics including age, gender, and dominant side. Secondly, small sample size and the presence of diverse fracture patterns may pose limitations in accurately defining a failure model and making predictions regarding future surgical interventions. Thirdly, we only analyzed radiographs taken immediately after operation and at 3-month follow-up, and did not include radiographs taken before the operation or at longer follow-up. However, we have confirmed that fractures of all patients healed, and the variation in our measurement was minimal after bone healing. Fourthly, no clinical functional outcomes, such as the MMWS and the DASH score, wrist range of motion, and grip strength, were documented in our study. This information could provide valuable insights into a more comprehensive assessment of the effectiveness of VAVLP fixation.

## 5. Conclusions

The use of VAVLP fixation has demonstrated the ability to sustain the stability of radiographic parameters including RH, UV, RI, VT, and TDA without significant reduction loss in the treatment of distal radius fractures classified as extra-articular or intra-articular. Tendon rupture risk can be minimized with this technique in both groups, with technical skill being crucial to achieving low risk. This risk can be maintained for at least three months, with the exception of complicated fracture cases. For intra-articular fractures treated with VAVLP fixation, the degree of subsequent displacement can be predicted using DDD measurements taken immediately after operation. However, further study is necessary to establish the predictive value of post-operative DDD values for the extra-articular fractures.

## Figures and Tables

**Table 1:** The characteristics of patients of two groups.

	Extra-Articular Group (N = 21)	Intra-Articular Group (N = 25)	*p*-Value
	Mean ± SD	Mean ± SD	
Age (year)	52.9 ± 18.5	54.0 ± 18.9	0.659 ^a^
Time of image taken after operation (Day)	1.0 ± 0.0	1.0 ± 0.0	1.000 ^a^
Time of 3 months follow-up image taken (Day)	83.3 ± 8.8	83.6 ± 13.1	0.956 ^a^
	N (%)	N (%)	
Dominant			0.551 ^b^
Right	7 (33.3)	11 (44.0)	
Left	14 (66.7)	14 (56.0)	
Gender			0.760 ^b^
Male	13 (61.9)	17 (68)	
Female	8 (38.1)	8 (32)	

^a^ = Mann–Whitney U test ^b^ = Fisher’s exact test.

**Table 2:** Comparison of radiographic parameters of the extra-articular group and intra-articular group immediately after the operation and 3-month following the operation.

			Extra-Articular Group	Intra-Articular Group	*p*-Value ^a^
RH	Post-OP	mean (mm)	10.75	11.40	
		SD (mm)	2.41	2.23	0.348
		range (mm)	7.27~15.15	7.11~15.58	
	3m-f	mean (mm)	10.80	11.35	
		SD (mm)	2.00	3.00	0.474
		range (mm)	6.48~14.51	3.16~17.28	
		*p*-value ^b^	0.899	0.926	
UV	Post-OP	mean (mm)	0.58	1.96	
		SD (mm)	2.32	2.53	0.063
		range (mm)	−4.05~4.22	−1.91~11.16	
	3m-f	mean (mm)	1.23	1.87	
		SD (mm)	2.05	2.05	0.300
		range (mm)	−3.76~4.41	−3.90~6.06	
		*p*-value	0.090 ^b^	0.503 ^c^	
RI	Post-OP	mean (°)	20.14	21.45	
		SD (°)	3.94	4.07	0.278
		range (°)	13.50~26.40	14.10~31.40	
	3m-f	mean (mm)	20.74	22.18	
		SD (mm)	3.95	5.46	0.320
		range (mm)	10.50~31.70	7.10~33.40	
		*p*-value ^c^	1.000	0.108	
VT	Post-OP	mean (°)	13.14	12.77	
		SD (°)	5.50	5.57	0.822
		range (°)	4.70~22.30	0.80~22.70	
	3m-f	mean (°)	13.60	12.80	
		SD (°)	5.69	4.94	0.614
		range (°)	2.40~24.40	4.40~21.90	
		*p*-value ^b^	0.521	0.963	
TDA	Post-OP	mean (°)	59.00	53.99	
		SD (°)	5.71	10.55	0.048
		range (°)	46.80~70.20	25.20~74.50	
	3m-f	mean (°)	56.67	53.06	
		SD (°)	9.14	8.63	0.176
		range (°)	33.00~68.80	35.00~69.30	
		*p*-value ^b^	0.274	0.551	
DDD	Post-OP	mean (°)	4.69	5.55	
		SD (°)	1.75	1.87	0.118
		range (°)	1.99~8.49	3.68~8.66	
	3m-f	mean (°)	4.71	4.77	
		SD (°)	1.66	1.58	0.893
		range (°)	1.83~8.82	2.53~9.01	
		*p*-value ^b^	0.956	0.008	

RH = Radial height, UV = Ulnar variance, RI = Radial inclination, VT = volar tilt, TDA = Tear drop angle. DDD = distal dorsal cortical distance. Post-op = estimated at post-operation immediately. 3m-f = estimated at 3 month follow-up. ^a^ = Student’s *t* test ^b^ = Paired *t* test ^c^ = Wilcoxon signed-rank test.

**Table 3:** Comparison of risk of tendon rupture by Soong classification at two check points and change of tendon rupture risk in 3 months between extra-articular and intra-articular group.

		Extra-Articular Group (N = 21)	Intra-Articular Group (N = 25)	*p*-Value
		N (%)	N (%)	
Risk of tendon rupture in post-op	Low risk	19 (90.5)	22 (88.0)	0.585 ^a^
	High risk	2 (9.5)	3 (12.0)	
Risk of tendon rupture in 3m-f	Low risk	19 (90.5)	24 (96)	0.443 ^a^
	High risk	2 (9.5)	1 (4.0)	
The change of risk in 3 months	Low risk to low risk	19 (90.5)	22 (88.0)	0.225 ^b^
	Low risk to high risk	0 (0.0)	2 (8.0)	
	High risk to high risk	2 (9.5)	1 (4.0)	

Post-op = estimated at post-operation immediately. 3m-f = estimated at 3 month follow-up. Low risk = Soong grade 0 and Soong grade 1. High risk = Soong grade 2. ^a^ = Fisher’s exact test ^b^ = likelihood Chi square test.

**Table 4:** Regression analysis of distal dorsal cortical distance of the two groups and two groups combined.

Variable		B	*p*-Value ^a^
Extra-articular group (N = 21)	Post-op Distal dorsal cortical distance	4.649	0.907
	C3m Distal dorsal cortical distance	0.052	
Intra-articular group (N = 25)	Post-op Distal dorsal cortical distance	4.519	0.031
	C3m Distal dorsal cortical distance	0.850	
Combined two groups (N = 46)	Post-op Distal dorsal cortical distance	4.561	0.048
	C3m Distal dorsal cortical distance	0.573	

Post-op = Radiographic parameters immediately after the operation. C3m = Radiographic parameters change in 3 months. ^a^ = Simple linear regression analysis.

## Data Availability

The datasets generated during the current study are available from the corresponding author on reasonable request.
